# *Corema album* Leaves Mediate DNA Damage in Triple-Negative Breast Cancer Cells

**DOI:** 10.3390/cimb44080246

**Published:** 2022-08-11

**Authors:** Ana Sofia Cerquido, Martin Vojtek, Rita Ribeiro-Oliveira, Salomé Gonçalves-Monteiro, Maria João Barroca, Aida Moreira da Silva, Olga Viegas, Victor Freitas, Joana Beatriz Sousa, Isabel M. P. L. V. O. Ferreira, Carmen Diniz

**Affiliations:** 1 LAQV/REQUIMTE, Laboratory of Pharmacology, Department of Drug Sciences, Faculty of Pharmacy, University of Porto, 4050-313 Porto, Portugal; 2 Unidade de I&D Química-Física Molecular, Department of Chemistry, University of Coimbra, 3004-535 Coimbra, Portugal; 3 Polytechnic of Coimbra, Coimbra Agriculture School, Bencata, 3045-601 Coimbra, Portugal; 4 LAQV/REQUIMTE, Laboratory of Bromatology and Hydrology, Department of Chemical Sciences, Faculty of Pharmacy, University of Porto, 4050-313 Porto, Portugal; 5 Faculty of Nutrition and Food Sciences, University of Porto, 4150-180 Porto, Portugal; 6 LAQV/REQUIMTE, Faculty of Sciences, University of Porto, 4169-007 Porto, Portugal

**Keywords:** natural product, maritime plant, anticancer, NF-κB pathway, ERK 1/2 pathway

## Abstract

*Corema (C.) album* is a shrub endemic to the Atlantic coast and has been described as yielding beneficial effects for human health. Nevertheless, studies concerning the bioactivity of *C. album* leaves are scarce. This study aims at investigating the anticancer potential and mode of action, of an hydroethanolic extract of *C. album* leaves (ECAL) on triple-negative breast cancer. This is a poor survival breast cancer subtype, owing to its high risk of distant reappearance, metastasis rates and the probability of relapse. The ECAL ability to prevent tumor progression through (i) the inhibition of cell proliferation (cell viability); (ii) the induction of apoptosis (morphological changes, TUNEL assay, caspase-3 cleaved) and (iii) the induction of DNA damage (PARP1 and γH2AX) with (iv) the involvement of NF-κB and of ERK1/2 pathways (AlphaScreen assay) was evaluated. ECAL activated the apoptotic pathway (through caspase-3) along with the inhibition of ERK and NF-κB pathways causing DNA damage and cell death. The large polyphenolic content of ECAL was presumed to be accountable for these effects. The extract of *C. album* leaves can target multiple pathways and, thus, can block more than one possible means of disease progression, evidencing the anticancer therapeutic potential from a plant source.

## 1. Introduction

Breast cancer, in 2020, is the second major cause of death in women worldwide, being the most commonly diagnosed female cancer type in several countries, including Portugal [1]. Triple-negative breast cancer (TNBC, a breast cancer subtype) is responsible for approximately 25% of all breast cancer cases [2] and usually occurs in women under the age of 40, in pre-menopause, being more frequent in women from Latin, African and African-American ethnicities [3]. TNBC is characterized by the lack of immunohistochemical expression of estrogen and progesterone receptors and HER2 overexpression [4] and has been associated with a higher risk of recurrence, metastasis and probability of relapse, with low survival rates compared to other breast cancer subtypes [2]. Indeed, 40% to 70% of TNBC patients with localized cancer will develop distant metastasis between the date of diagnosis and the following five years [4]. Hence, novel drugs, having antiproliferative or anti-invasive properties, can become relevant to fight cancer, including the resistant subtypes. Plant-derived products still have a huge potential to provide novel drugs since their natural compounds may target and disrupt different vias involved in cancer progression [5] and may also contribute to overcoming cancer resistance [5]. Thus, the screening of non-well studied singular plants is on demand, and one example is the Iberian plant *Corema (C.) album*.

*C. album* belongs to the family Ericaceae and is a shrub endemic to the Atlantic coast of the Iberian Peninsula, although it can also be found in other countries [6], and is one of the medicinal plants included in the herbarium of Francesc Bolòs [7]. *C. album* presents sexual dimorphism, and its fruits are berries produced by the female plants and ripen in early summer (June and July) in the south and a little later (August and September) in the north [8]. *C. album* fruits have been used as food or food ingredient for many centuries, fresh as appetizers and juices and can also be used to make jams [9]. In addition, these fruits have been employed in traditional medicine to treat fever and intestinal pinworm infection [7,8,10]. *C. album* berries contain polyphenolic compounds that attracted scientific community attention regarding its health beneficial properties [11] ranging from prevention of cardiovascular and/or neurodegenerative diseases to protection against oxidative stress, inflammation and cancer [12]. In addition to the fruits, studies concerning *C. album* leaves regarding its anticancer effects in human are limited to 2′,4′-dihydroxydihydrochalcone, 2′-methoxy-4′-hydroxydihydrochalcone, 2′,4′-dihydroxychalcone and pinocembrin compounds [13,14] identified in ethyl acetate extracts. The goal of the present study is to investigate the effects, and possible mechanisms of action, of a hydroethanolic extract of *C. album* leaves (ECAL) on TNBC using MDA-MB-231 cells.

## 2. Materials and Methods

### 2.1. Chemicals and Reagents

Fetal bovine serum (FBS; EU Approved) was obtained from Gibco (Gibco/Life Technologies, Paisley, UK). Dulbecco’s modified Eagle’s high glucose medium (DMEM-HG), Dulbecco’s modified Eagle’s medium and Ham’s F12 cell growth medium (DMEM/F12), horse serum, human epidermal growth factor (hEGF), bovine insulin, hydrocortisone, penicillin-streptomycin, trypsin, paraformaldehyde, Hoechst 33258 and Angiotensin II were purchased from Sigma (Sintra, Portugal).

In Situ Cell Death Detection Kit, TMR red (Roche Diagnostics GmbH, Mannheim, Germany), rabbit anti-caspase-3 cleaved (Abcam, London, UK), rabbit anti-PARP1 cleaved (Abcam, London, UK), rabbit anti-γH2AX (Cell Signaling Technology, Danvers, MA, USA) were used. AlphaScreen SureFire ERK1/2 & pERK1/2 Kit and AlphaScreen SureFire NF-κB & pNF-κB Kit were purchased from PerkinElmer (I.L.C., Lisboa, Portugal). Viscozyme^®^ was from Novozymes (Bagsvaerd, Denmark). Other reagents were of analytical grade. Ethanol concentration in the culture medium did not exceed 0.1% (*v*/*v*) and was considered as the control.

### 2.2. Preparation of Extract of C. album Leaves (ECAL)

The leaves of the *Corema album* were collected in a specific area granted to scientific research near the city of Ovar (GPS: 40.42019° N, 8.76424° W, Portugal) during the summer of 2019 (from June till August). After the harvest, leaves were immediately separated from the stems and thoroughly cleaned. The extract prepared with fresh leaves was carried out by adapting the methods of Zhao et al. [15]. Briefly, fresh *C. album* leaves were washed with tap water and shredded using an industrial homogenizer (Retsch GM 200), resulting in a paste. Next, 3 samples containing 1 g of paste and one blank with 1 mL of water were prepared in 50 mL tubes, followed by the addition of 20 mL of distilled water. The pH values were adjusted to pH 5.5. After this, 1.8 mL of Viscozyme^®^ (cell wall degrading enzyme complex from *Aspergillus* sp.) were added and the samples were homogenized. Next, the samples were incubated in a water bath for 3 h at 45 °C and every 30 min the tubes were agitated. After the incubation period, 400 mL of ethanol 96% were added to each sample, homogenized and left to rest overnight. The day after, the ethanolic solution was transferred to tubes and centrifuged (3500× *g*, 30 min). The fractions were separated, and the supernatant was kept at −20 °C until evaporation and lyophilization. The supernatant was evaporated using a rotor evaporator, followed by free-drying in order to obtain a powdered extract that was kept at −20 °C until analysis.

### 2.3. Quantification of Total Phenolic Content

The total phenolic content was determined through the Folin–Ciocalteu (F–C) method. Briefly, the powdered ECAL was dissolved in absolute ethanol and water (2:1). For quantification, samples were then diluted with water (1:100). In a 96-well plate, samples were successively diluted and analyzed with F–C reagent (4/10 *v*/*v*) in triplicate and the evaluation of the intrinsic absorption of the ECAL were also tested using water instead of Folin. After the addition of the F–C reagent, the microplate was shaken in the microplate reader for 1 min before 10 min of incubation. Subsequently, NaCO_3_ (6% *m*/*v*) was added to every well. The consequent reduction of the phosphomolybdate-phosphotungstate complex in alkaline media operated by the phenolic compounds was measured, at 765 nm, after 120 min using a Synergy HT plate reader from BioTek (Winooski, VT, USA). Gallic acid 1 mM was used as standard (31.25–500 µM). Results were expressed in milligrams of gallic acid equivalents per gram (mg GAE/g).

### 2.4. HPLC Analysis and Mass Spectrometry Analysis of Phenolic Content of ECAL

ECAL was analyzed by HPLC-DAD/MS. A Finnigan Surveyor series liquid chromatograph, equipped with a Thermo Finnigan (Hypersil Gold) reversed-phase column, (150 mm × 4.6 mm, 5 μm, C18) thermostated at 25 °C, was used. Two solvents were employed for elution: A—water/acetic acid (99:1; *v*/*v*), and B—water/acetonitrile/acetic acid (79:20:1; *v*/*v*/*v*). The gradient profile was: 0 to 55 min, 80% to 20% A; 55 to 70 min, 20% to 10% A and 70 to 90 min, 10% to 0% A, at a flow rate of 0.3 mLmin-1. The sample injection volume was 20 μL. The chromatographic column was washed with 100% B for 10 min and then stabilized with the initial conditions for another 10 min. Double-online detection was carried out by a photodiode spectrophotometer and mass spectrometry. The mass detector was a Finnigan LCQ DECA XP MAX (Finnigan Corp., San Jose, CA, USA) quadrupole ion trap equipped with atmospheric pressure ionization (API) source, using electrospray ionization (ESI) interface. The vaporizer and the capillary voltages were 5 kV and 4 V, respectively. The capillary temperature was set at 325 °C. Nitrogen was used as both sheath and auxiliary gas at flow rates of 80 and 30, respectively (in arbitrary units). Spectra were recorded in negative ion mode between *m*/*z* 120 and 2000. The mass spectrometer was programmed to do a series of three scans: a full mass, a zoom scan of the most intense ion in the first scan and a MS–MS of the most intense ion using relative collision energies of 30 and 60 V.

### 2.5. Cells Culture

MDA-MB-231 cells (human Caucasian triple negative (ER−, PR−, Her2−), claudin-low breast carcinoma, ATCC HTB-26, from ATCC (Manassas, VA, USA)) were cultured in DMEM-HG cell growth medium supplemented with 10% (*v*/*v*) FBS. MCF-12A cells (non-neoplastic breast cell line, ATCC CRL-10782, from ATCC (Manassas, VA, USA)) were cultured in DMEM/F12 medium supplemented with 20 ng/mL hEGF, 100 ng/mL cholera toxin, 0.01 mg/mL bovine insulin, 500 ng/mL hydrocortisone and 5% (*v*/*v*) horse serum. Cells were cultured in monolayers in a sterile environment at 37 °C with 5% CO_2_ humidified atmosphere. Under these conditions the population doubling time was 25.5 ± 0.9 h and 20.6 ± 3.1 h for MDA-MB-231 and MCF-12A cells, respectively. The cell cultures were routinely screened for mycoplasma contamination presenting negative results. Tested compounds were added during the logarithmic phase of cell growth.

### 2.6. Cell Viability

Cells were seeded in 96-well microplates (1.5 × 10^4^ cells/cm^2^) and left for 24 h to attach. Thereafter, the growth medium was replaced with a growth medium containing ECAL (1.7–1730 μg/mL). The label-free kinetic live monitoring of cell proliferation was performed using a LionheartFX automated microscope (BioTek, Winooski, VT, USA) with the direct image acquisition of cells in microplates at 0, 24, 48 and 72 h of the post-addition of the tested compounds. Acquired 4X images were processed using Gen 5 Image Analysis software (BioTek, Winooski, VT, USA), which allows the identification and counting of individual cells per image. The normalized cell growth (%) was calculated using the following formula: C(*t*) = [(Number of Treated Cells(*t*) − Number of Treated cells(0))/(Number of Untreated Cells(*t*) − Number of Untreated Cells(0))] × 100. Where C(*t*) is the percent of net cell growth over time, Number of Treated Cells(t) is the count of cells treated with drug at each time point, Number of Treated cells(0) is the count cells treated with drug at time 0 h, Number of Untreated Cells(*t*) is the count of untreated (control) cells at each time point and Number of Untreated Cells(0) is the count of untreated (control) cells at time 0 h.

### 2.7. Cell Morphology

MDA-MB-231 or MCF-12A cells were grown and their morphological changes in response to 24 h treatment with ECAL (1.7–1730 μg/mL) were acquired using a phase contrast objective (20×). Cell nuclei were stained with Hoechst 33258 (5 μg/mL) for 2 min in the dark. Cells treated with vehicle (growth media containing 0.26% ethanol) served as an appropriate control. Finally, mounting medium (glycerol/PBS) was added directly to the wells. Cell micrographs were immediately captured with a 20× objective of contrast phase or fluorescence using the Lionheart™ FX Automated Live Cell Imager (BioTek, Winooski, VT, USA). Image analysis was performed using Gen5 Image Prime 3.02 (Biotek, Winooski, VT, USA).

### 2.8. Tunel Assay

Terminal deoxynucleotidyl transferase-mediated dUTP nick end labeling (TUNEL) DNA fragmentation, a characteristic of apoptosis, was assessed using an In Situ Cell Death Detection Kit, TMR red, following the manufacturer’s instructions. Briefly, breast cancer cells (5 × 10^4^) were cultured on 6-well plates over coverslips and incubated for 24 h. The cells were treated with increasing concentrations of ECAL (1.7–1730 μg/mL) and media alone for 24 h. Additionally, cells were treated with DNAse (3000 U/mL) for 5 min. Next, the cells were fixed in paraformaldehyde solution. The cells were incubated in a permeabilization solution (0.1% Triton X-100 in 0.1% sodium citrate) for 2 min in ice and then treated with a mixture of label solution and enzyme solution for 1 h in the dark at 37 °C. Cell micrographs were immediately captured with 20× objective using The Lionheart™ FX Automated Live Cell Imager (BioTek, Winooski, VT, USA). Image analysis was performed using Gen5 Image Prime 3.02 (Biotek, Winooski, VT, USA).

### 2.9. Western-Blot Analysis

Cells were seeded in 6-well plates (1.5 × 10^4^ cells/cm^2^) and left for 24 h to attach. Afterwards, cells were exposed to increasing concentrations of ECAL (1.7–1730 μg/mL) for 48 h. Cells were washed with PBS and lysed with RIPA buffer supplemented with protease inhibitors cocktail (Abcam, UK). Protein concentration was determined using the Bradford assay. Samples (50 µg of proteins in 6X Laemmli sample loading buffer) were separated by SDS–PAGE using 12% TGX Stain-free gels (Bio-Rad, Lisbon, Portugal) and blotted onto PVDF membrane. Membranes were blocked with 5% BSA, during 1 h at room temperature (RT) and probed with rabbit anti-cleaved caspase-3 (1:500), rabbit anti-γH2AX (1:1000) and rabbit anti-cleaved PARP-1 (1:1000) antibodies overnight at 4 °C. Blots were washed with TBST, incubated with goat anti-rabbit horseradish peroxidase (HRP) conjugated secondary antibody (1:5000) during 1 h, at RT, washed with TBST and the immunocomplexes were detected using the Novex ECL Chemiluminescent kit (Invitrogen, Life Technologies, Madrid, Spain) and ChemiDoc MP Imaging System (Bio-Rad, Lisbon, Portugal). Chemiluminescent signal was quantified using ChemiDoc™ Imaging Systems with Image Lab™ Software Instrument (Bio-Rad, Lisbon, Portugal). The stain-free signal of total protein per lane was also captured and used for the normalization of immunolabeled protein bands relative to the total proteins in the respective lane.

### 2.10. ERK1/2 Phosphorylation Assay

Changes in ERK1/2 phosphorylation (pERK) were detected using the AlphaScreen SureFire ERK1/2/pERK1/2 Kit. The assay was performed according to the manufacturer’s instructions. Briefly, cells were treated with increasing concentrations of ECAL (1.7–1730 μg/mL) for 1 h or with 300 nM of Ang II (as a positive control) for 15 min at 37 °C. The medium was aspirated, cells were lysed in 40 μL lysis buffer and cell lysates (10 μL) were transferred to 384-well ProxiPlates (PerkinElmer-I.L.C., Lisboa, Portugal) for detection. Data are expressed relative to basal.

### 2.11. NF-κB Phosphorylation Assay

Changes in NF-κB phosphorylation were detected using the AlphaScreen SureFire NF-κB/pNF-κB Kit. The assay was performed according to the manufacturer’s instructions. Briefly, cells were treated with increasing concentrations of ECAL (1.7–1730 μg/mL) for 1 h or with 300 nM of Ang II (as a positive control) for 15 min at 37 °C. Medium was aspirated, cells were lysed in 40 μL lysis buffer and cell lysates (10 μL) were transferred to 384-well ProxiPlates (PerkinElmer-I.L.C., Lisboa, Portugal) for detection. Data are expressed relative to basal.

### 2.12. Statistical Analysis

Results are means ± standard error of the mean (SEM) and are expressed as a percentage of control of *n* independent experiments performed. One-way ANOVA followed by post hoc Dunnett’s multiple comparisons test or Holm–Sidak’s multiple comparison test was performed using GraphPad Prism version 7.0 for Windows (GraphPad Software, La Jolla, CA, USA). A *p*-value lower than 0.05 was considered to denote statistically significant differences.

## 3. Results

### 3.1. ECAL Supresses the Proliferation of Triple-Negative Breast Cancer

**Figure 1:**
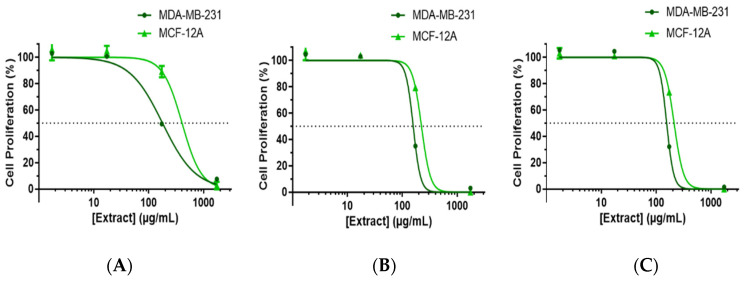
Inhibition of cell proliferation induced by hydroethanolic extract of *C. album* leaves (ECAL) in MDA-MB-231 and MCF-12A cells. Cells were treated with increasing concentrations of ECAL (1.7–1730 μg/mL) for (**A**) 24 h, (**B**) 48 h and (**C**) 72 h. Label-free kinetic live monitoring of cell proliferation was performed using a LionheartFX automated microscope with the direct image acquisition of cells in microplates at 0, 24, 48 and 72 h of the post-addition of the tested compounds.

The ECAL-induced suppression of MDA-MB-231 cell proliferation after 24, 48 and 72 h of incubation within a concentration range between 1.7–1730 μg/mL in a concentration-dependent manner, as shown in Figure 1. 

To observe a potential selective effect, the ECAL was also studied on healthy breast cells (MCF-12A): ECAL incubated for 24 h revealed to suppress MCF-12A cells proliferation only with concentrations above approximately 100 μg/mL. In fact, the IC50 (24 h) for MCF-12A was 223 μg/mL, i.e., 1.4 higher than that observed for MDA-MB-231 (Figure 1A). For longer incubation periods (48 h or 72 h), the difference in the concentration–response curve for the two cell lines in study is attenuated; however, the IC50 (48 h or 72 h) for MDA-MB-231 is still lower compared to MCF-12A values (158.1 or 155.2 compared to 220.6 or 211.1, respectively—Figure 1B or Figure 1C).

**Figure 2:**
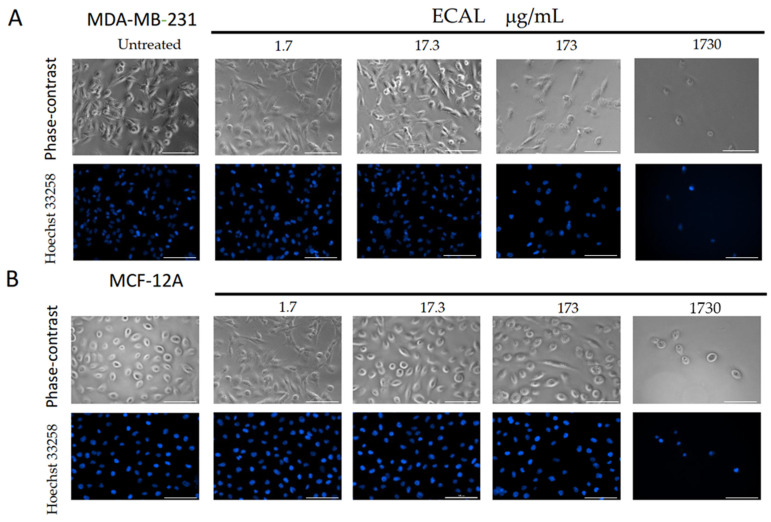
Morphological changes induced by increasing concentrations of the hydroethanolic extract of *C. album* leaves (ECAL) (1.7–1730 μg/mL) incubated for 24 h in (**A**) MDA-MB-231 cells or (**B**) MCF-12A cells. Upper panel—representative images from five independent experiments obtained under an objective lens of a phase-contrast of the Lionheart microscope; lower panel—representative images from five independent experiments with nuclei stained with Hoechst 33258 (blue) obtained under an objective lens of a Lionheart microscope. Scale bar = 100 μM.

The microscopic observation of MDA-MB-231 cells treated with increasing concentrations of ECAL revealed various morphological changes, including membrane blebbing, a low cell density and more floating cells (Figure 2A,B, upper panel). To verify whether the inhibition of cell proliferation by ECAL could be linked to apoptosis, nuclear morphological changes were also evaluated. In ECAL-treated cells, nuclear fragmentation and chromatin condensation was observed (Figure 2A,B, lower panel).

### 3.2. ECAL Induced Double-Stranded DNA Damage and Apoptosis via Caspase 3

**Figure 3:**
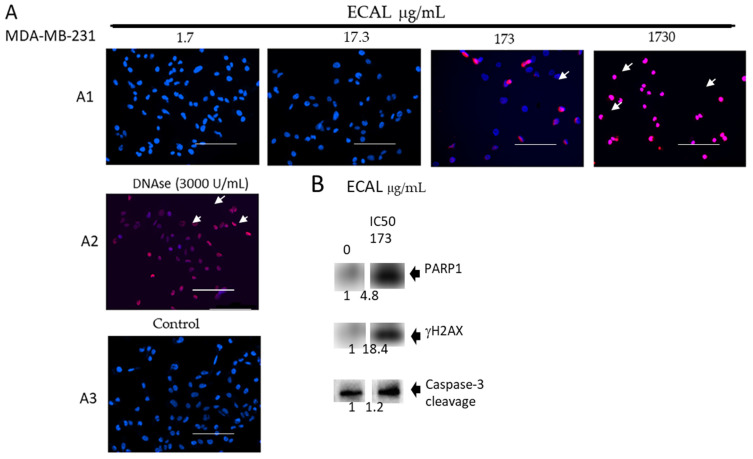
DNA damage induced by the hydroethanolic extract of *C. album* leaves (ECAL). (**A**) TUNEL staining for apoptosis in MDA-MB-231 cells incubated for 24 h. (**A1**) with the increasing concentrations of ECAL (1.7–1730 μg/mL); (**A2**) with DNAse (3000 U/mL); (**A3**) without treatment (control). Blue nuclear stain and green TUNEL stain. TUNEL-positive apoptotic cells (arrows) are those in which the green and blue colocalize to the same cell. Scale bar = 100 μM; (**B**) immunoblot images of proteins γH2AX, PARP1 and Caspase-3 cleaved (histone H3, as the internal control) wherein the fold changes have been presented below each panel.

To confirm the occurrence of apoptosis (suggested by data observed in Figure 2), double stranded DNA fragmentation was evaluated by TUNEL assay in ECAL-treated MDA-MB-231 cells (Figure 3A). Results of the increasing concentrations of ECAL (1.7–1730 μg/mL) showed that DNA fragmentation occurred with 173 μg/mL, after 24 h of incubation. Cancer cells exposed to DNAse (used as a positive control) were also positively stained, as expected. As a control, cancer cells treated with solvent (control) or only with medium (not shown) did not exhibit fluorescence staining compatible with apoptotic cells. Cleaved caspase-3 expression (apoptosis marker) increased in ECAL-treated cells (Figure 3B). These results are compatible with ECAL-induced cell death by apoptosis instead of necrosis in MDA-MB-231 cells.

Further investigation revealed accumulation of double-stranded DNA breaks since phosphorylated histone γH2A.X (Ser139) increased in response to the treatment of cells with ECAL: a near 20-fold upregulation for γH2AX (Figure 3B). Additionally, PARP1 (a DNA damage marker) expression also increases near 5-fold relative to the control.

**Figure 4:**
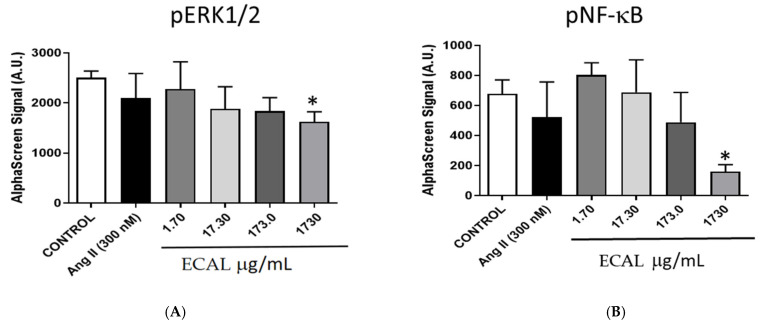
Hydroethanolic extract of *C. album* leaves (ECAL) effects on ERK and NF-kB pathways in MDA-MB-231 cells. (**A**) Phosphorylated ERK1/2 and (**B**) Phosphorylated NF-κB, measured in the presence of negative control, positive control (angiotensin II (Ang II), 300 nM) and with increasing concentrations of ECAL (1.7–1730 μg/mL). Significant differences from the values obtained in control: * *p* < 0.05 (One-way ANOVA followed by post hoc Dunnett’s multicomparison *t*-test.

MDA-MB-231 breast cancer cells evidenced high endogenous extracellular signal-regulated kinase 1/2 (ERK1/2) phosphorylation [16]; thus, we investigated the effect of ECAL on ERK activity using AlphaScreen technology. Angiotensin II (Ang II), which stimulates the ERK1/2 pathway, did not further increase pERK1/2. Results showed a progressive downregulation of phosphorylated ERK1/2 in the presence of increasing concentrations of ECAL (1.7–1730 μg/mL) (Figure 4A). A significant difference was observed for the highest concentration tested.

Nuclear factor kappa-B (NF-κB) has been described to be involved in the blockage of apoptosis and cell proliferation [17]. Thus, we evaluated the ability of ECAL to suppress the activation of NF-κB. ECAL showed an inhibition of total NF-κB compared to values observed in control MDA-MB-231 cells (data not shown). In addition, the phosphorylated NF-κB measurement demonstrated that increasing concentrations of ECAL (1.7–1730 μg/mL) downregulated the phosphorylated forms of NF-κB, which are known to be involved in the translocation to nucleus.

Taken together, these data support the inhibition of cell proliferation and apoptosis by ECAL as the major demonstration of the possible mode of action of ECAL.

### 3.3. Polyphenolic Content of ECAL

The extract prepared with *C. album* leaves had a total phenolic content of 67.1 ± 4.0 mg GAE/g DW. ECAL revealed a wide variety of phenolic compounds belonging to different subclasses: hydroxybenzoic acids, hydroxycinnamic acids, flavanols and flavonols (Table 1). Some of the phenolic compounds identified in ECAL are bound to carbohydrates, enhancing their complexity. 

## 4. Discussion

This study reveals, for the first time, the anticancer therapeutic potential of a plant source, *C. album* (as extract of leaves, ECAL) towards TNBC. The study described multiple pathways modulated by the leaves extract. ECAL activated the apoptotic pathway (through caspase-3) along with the inhibition of ERK and NF-κB pathways causing DNA damage and cell death. Targeting multiple pathways indicates that ECAL can block more than one possible means of disease progression, which is a signature of a good anticancer drug.

The knowledge regarding the DNA damage response and its relation to carcinogenesis has increased in the last decade, revealing multiple pathways that can be modulated to prevent or treat cancer [18]. Plant derived compounds may target and disrupt more than one of these pathways [5]; thus, having anticancer effects and may also contribute to overcome cancer resistance [5]. In the current study, ECAL is revealed to be able to suppress TNBC proliferation in a concentration-dependent manner (24 h of incubation). For longer incubation periods, the impact on cell proliferation occurs only for higher concentrations of ECAL. Although ECAL evidenced cytotoxicity also for non-neoplastic cells (MCF-12A), the amount of extract needed to cause similar cytotoxicity in MCF-12A cells is higher. Moreover, when the concentration used is the IC50 for MDA-MB-231 (24 h of incubation with ECAL), the proliferation of MCF-12A cells is not affected. Such a result reveals that ECAL presents some selectivity towards TNBC. The anticancer properties herein reported for breast cancer, and particularly for TNBC, are in agreement with the reported anticancer effects of *C. album* in other cancer types: in human colon carcinoma cells, HT-29 (leaves: [13,19]), as well as in human intestinal epithelium cells, Caco-2 cells (juice form berries: [20]).

TNBC treated with ECAL yielded morphological cell changes (Figure 2) with apoptotic features, such as nuclear condensation, apoptotic body formations and DNA fragmentation (Figure 2 and Figure 3). Such changes are suggestive that ECAL induces apoptosis. Apoptosis is one of the major death mechanisms associated with the cytotoxicity of anticancer drugs, removing the malignant cells without damaging the healthy cells. Generally, apoptosis is divided into extrinsic and intrinsic pathways [21]: extrinsic pathway is usually considered to be initiated by death receptor binding to ligands, activating caspase-8, which in turn directly activates caspase-3 (an effector caspase), promoting cell degradation [21]; intrinsic or mitochondrial pathways can be triggered by diverse stimuli and recent reports indicates caspase-2 as the most apical caspase [22] in the caspase cascade, while caspase-9 seems to have a key role by promoting caspase-3 cleavage [21]. Caspase-3 is known to be cleaved at an aspartate residue generating a p12 and a p17 subunit, the active caspase-3 enzyme [23]. Cleaved caspase-3 can degrade a variety of cellular proteins, being also responsible for morphological changes and DNA fragmentation during cell apoptosis [24,25]). In the present study, cleaved caspase-3 expression in ECAL-treated cells was evaluated, demonstrating an upregulation. This finding is suggestive that ECAL induces apoptosis through a caspase-3-dependent pathway in TNBC.

The activation of effector caspases, such as caspase-3, may degrade several substrate proteins, such as PARP1, preventing DNA repair-induced survival. In the present study, an increased expression of cleaved PARP1 in ECAL-treated cells was found (Figure 3B), suggesting poor survival in TNBC. PARP1 cleavage has been described in response to a variety of anticancer drugs functioning as DNA interstrand cross-linkers [21,26]. It has been described that when cells are submitted to DNA-damaging chemotherapeutic agents, namely to cisplatin or doxorubicin, double-stranded breaks are produced, rapidly resulting in the phosphorylation of histone H2AX at Ser 139 (γH2AX) [27]. γH2AX is associated to DNA damages (revealed increase in γH2AX), which are known to be able to trigger intrinsic apoptosis [10.1007/s00018-013-1555-2]. In the current study, γH2AX evidenced the double-stranded DNA damage in ECAL treated cells (Figure 3B). In accordance with these data other plant extracts studies have reported the induction of the expression of apoptotic proteins (*Menyanthes trifoliata* L.: [28]; *Elaeagnus angustifolia:* [29]; *Asteraceae Brachylaena*: [30]) and of DNA damage (*Aronia melanocarpa, Cornus mas,* and *Chaenomeles superba*: [31]; *Echinodorus macrophyllus*: [32]; *Tiliacora racemose*: [33]). 

The extrinsic apoptotic pathway is regulated, among other processes, by a nuclear transcriptional factor, the NF-κB, which modulates the expression of several genes and of several proteins involved in the apoptotic process [34]. For example, the inhibition of NF-kB lead to a reduction of IAPs [33], such as Survinin [35], which are known to be involved in the inhibition of apoptosis, namely by inhibiting caspase-9 and, thus, indirectly facilitating the activation of caspases and apoptosis [25]. Accordingly, the results presented in the current study are in line with these findings, evidencing a downregulation of the expression of phosphorylated NF-κB along with an increased expression of cleaved caspase-3 (Figure 3) in TNBC treated with ECAL. In several other cancer types, similar evidence regarding the association between a downregulation expression of NF-κB and apoptosis, such as in prostatic cancer (LNCaP cells: [36]; PC3 cells: [37]), pancreatic carcinoma cells, fibrosarcoma and colorectal cancer cells [38,39,40]. A reduction of NF-κB activation was also found to occur with other medicinal plants [41] and, thus, counteracting the previous reported NF-κB-induced apoptosis resistance that commonly occurs in cancer treatment [41]. This notwithstanding, the activation of NF-κB can be regulated by PARP1 [42]: PARP1 synergistically co-activates NF-κB-dependent gene expression by interacting with histone–acetyl transferase and CREB binding protein. Such interactions promote pro-inflammatory cytokines. PARP1 cleavage observed in response to ECAL treatment might attenuate and, therefore, upregulate these cytokines. 

The downregulation of the ERK1/2 pathway by ECAL is also in accordance with the hypothesis of the ECAL induction of apoptosis in TNBC. In previous studies, it has been demonstrated that the occurrence of a constitutive activation of the ERK pathway in MDA-MB-231 cells [16] regulated transcription factors, protein kinases and phosphatases, the regulators of apoptosis, along with other signaling molecules [43]. The ERK1/2 pathway has been implicated in signaling and may crosstalk with other signaling pathways, any change in ERK1/2 activation will have, therefore, an important impact in these malignant cells. In fact, the inhibition of this pathway has previously been associated with apoptosis induction in cervical cancer [44], colon cancer [45], pancreatic stellate cells [46], oral cancer [47] and other cancers [48], and thus, this pathway constitutes an important target in the fight against cancer. In accordance, the ERK1/2 inhibitor, Ulixertinib (BVD-523, VRT752271), has recently entered into clinical studies for the treatment of advanced solid tumors and pancreatic cancers, among others [49]. 

The literature concerning the bioactive phytochemicals identified in *C. album* leaves is scarce [50]. Hydroethanolic extracts contain flavonol derivates, especially myricetin, (epi)catechin and proanthocyanidins [51], whereas the flavonoids, namely 2′,4′-dihydroxydihydrochalcone, 2′-methoxy-4′-hydroxydihydrochalcone, 2′,4′-dihydroxychalcone [13,14] are also present. Previous studies have also reported the capability of flavonoids to be chemo-preventive [21,49], to be able to reduce cell proliferation and to induce intracellular events that lead to cell cycle arrest, apoptosis and DNA injury, namely in TNBC [52]. The analysis of phenolic compounds in ECAL revealed a diverse profile of compounds (Table 1), being closer with the profile described on hydroethanolic extracts [51]. ECAL was revealed to be rich in phenolic acids, namely in hydroxybenzoic and hydroxycinnamic acids, and in flavonoids, particularly in flavones and flavonols: all of them are described previously as caspase activators [53]. Moreover, flavonols and flavones were also linked to PARP cleavage [21] promotion [53]. Under these subclasses, compounds such as catechin, epicatechin and quercetin were identified in ECAL, whose health benefits include the prevention of cardiovascular diseases, diabetes, neurodegenerative disorders and cancer, mainly through their antioxidant and anti-inflammatory properties [53]. Hence, it is conceivable that the anticancer properties of *C. album* leaves shown in this study can be explained, at least in part, by their polyphenolic composition, although the contribution exerted by chalcones, as intermediates in the biosynthesis of flavonoids and isoflavonoids, should be also taken into consideration.

## 5. Conclusions

This study revealed, for the first time, that that ECAL (the hydroethanolic extract of *C. album* leaves) has potent anticancer properties against TNBC, with a glimpse on its mode of action. ECAL revealed an enriched fraction of phenolic compounds, namely of phenolic acids and flavonoids as the putative responsible compounds involved in the modulation of multiple pathways: the activation of apoptosis through caspase-3 along with the inhibition of ERK and NF-κB pathways causing DNA damage and cell death. This capability to target multiple pathways is an important indicator that ECAL can block more than one possible means of cancer progression, which is a signature of a good anticancer drug. 

## Figures and Tables

**Table 1:** Phenolic compounds tentatively identified in ECAL by mass spectrometry.

Class	Compound	MW
**Hydroxybenzoic acids**	4-Hydroxybenzoic acid hexoside	300
	Protocatechuic acid hexoside	316
**Hydroxycinnamic acids**	Coumaroyl glucose	326
	Sinapoyl glucose	386
	Vanillyl glucose	330
	Feruloyl glucose	356
**Flavanols**	(+)-Catechin	29,068
	(−)-Epicatechin	290,268
	(+)-Catechin 3-O-glucose	452,409
	Procyanidin dimer type A	576
	Procyanidin trimer type A	864
	Procyanidin trimer type A	862
	Procyanidin tetramer type A	1152
	Procyanidin tetramer type A	1150
	Procyanidin pentamer type A	1440
	Procyanidin galhate	880
**Flavones or Flavonols**	Myricetin hexoside	480
	Myricetin dihexoside	642
	Rhamnetin hexoside	478
	Quercetin rhamnosyl hexoside	610
	Quercetin hexoside	464
	Kaempherol hexoside	448
	Myricetin methyl ether hexoside	494
	Myricetin xyloside	450

## Data Availability

The data presented in this study are available on request.
